# Analytical consideration of liquid droplet impingement on solid surfaces

**DOI:** 10.1038/s41598-017-02450-4

**Published:** 2017-05-24

**Authors:** Yukihiro Yonemoto, Tomoaki Kunugi

**Affiliations:** 1 Priority Organization for Innovation and Excellence, Kumamoto University, 2-39-1, Kurokami, Chuo-ku, Kumamoto-shi, Kumamoto, 860-8555 Japan; 2 Department of Nuclear Engineering, Kyoto University, C3-d2S06, Kyoto Daigaku-Katsura, Nishikyo-ku, Kyoto, 615-8540 Japan

## Abstract

In industrial applications involving spray-cooling, combustion, and so on, prediction of the maximum spreading diameter of a droplet impinging on a solid surface permits a quantitative estimation of heat removal and energy consumption. However, although there are many experimental studies regarding droplet impingement behaviour, theoretical models have an applicability limit for predicting the maximum spreading diameter. In the present study, we have developed an analytical model for droplet impingement based on energy conservation that considers adhesion energy in both horizontal and vertical directions at the contact line. The theory is validated by our experiment and existing experimental data possessing a wide range of Weber numbers. We demonstrate that our model can predict *β*_m_ (*i*.*e*., the maximum spreading diameter normalised in terms of initial droplet diameter) for various Newtonian liquids ranging from micro- to millimetre-sized droplets on different solid surfaces and can determine the transition between capillary and viscous regimes. Furthermore, theoretical relations for scaling laws observed by many researchers are derived.

## Introduction

Droplet impingement on solid surfaces is of great importance to ink-jet printing^1^, spray-cooling^2, 3^, combustion^4^, and coating processes^5^. The physics of droplet impingement are related not only to fluid dynamics but also to the respective interfacial properties of the liquid and solid. Droplet impingement is especially important for spray-cooling and combustion applications, whereby heat transfer from solid surfaces to droplets influences their impingement behaviour. This gives rise to key problems such as surface coverage efficiency and reducing fuel or coolant consumption. Quantitative estimation of heat transfer between the solid and the liquid film resulting from droplet impingement is important for the design of an efficient heat exchanger. The diameter of this film is mainly characterised by the maximum spreading diameter. In other applications, such as pesticide deposition on plant leaves, it is important to achieve maximum coverage of the target materials with the minimum amount of liquid. Therefore, the maximum spreading diameter is considered to be the most important factor for droplet impingement on solid surfaces.

When a droplet impinges on a solid surface, it spreads rapidly in a radial fashion. The maximum spreading diameter (*d*_max_) depends on the impinging velocity of the droplet, as well as the properties of the liquid and solid. This behaviour is mainly governed by the inertia of the droplet and the capillary and viscous forces. After the impingement, the spreading droplet breaks up if the capillary force is weak and the inertia dominates^6^. These forces are expressed by non-dimensional numbers; namely the Weber number, We(We = *ρ*_l_ *u* ^2^ *d*_0_/*σ*_lg_; where *ρ*_l_, *u*, *d*_0_ and *σ*_lg_ represent the liquid density, impinging velocity, initial droplet diameter and surface energy density of the liquid, respectively), and the Reynolds number, Re (Re = *ρ*_l_ *ud*_0_/*μ*_l_; where *μ*_l_ is the viscosity of the liquid). When considering droplet impingement behaviour, the maximum spreading diameter of a droplet is typically normalised with respect to its initial diameter (*d*_0_) as the dimensionless maximum spreading diameter, *β*_m_ = *d*_max_/*d*_0_^7–10^. Subsequently, the breakup behaviour of the droplet impinging on the solid surface is mainly discussed using Re and We to identify the thresholds between the spreading and the breakup behaviours^7, 11–14^. To understand the detailed mechanism for the droplet impingement on the solid surfaces, it is important to know the critical condition whereupon the droplet breaks up. However, even the spreading process before reaching the critical condition is still not completely understood. Therefore, the present study only focuses on the spreading behaviour of the droplet before the breakup process.

Current knowledge of the detailed behaviour of droplet impingement derived from experimental studies has improved gradually with advancements in high-speed video technology^15–24^. Theoretical approaches, on the other hand, employ models that attempt to predict the maximum spreading diameter of droplet impingement based on energy balance, momentum balance, and empirical considerations^7, 9, 10, 25–27^. Existing models can be classified into two main categories: 1) *β*_m_ as a function of Re and We (or a single parameter of Re or We)^2, 17, 19, 22, 26–28^ and 2) *β*_m_ as a function of Re, We, and cos *θ*_d_,^9, 10, 25^, where *θ*_d_ is the dynamic contact angle (advancing contact angle). It is only in the latter case that the interactions between the solid and liquid are considered. Due to the difficulty in predicting *θ*_d_ theoretically, however, experimental data is often used instead. Although the studies found in the literature have generally reported scaling laws of *β*_m_ ∝ We^1/2^ in the capillary region and *β*_m_ ∝ Re^1/5^ in the viscous region^7^, most models have an applicability limit in the extent of the We (or Re) number for predicting the experimental data. Therefore, the quantitative prediction of *β*_m_ for a wide range of We (or Re) numbers is indeed a challenging problem, especially since there are few models that can accurately predict solid surface properties. Thus, the important open question that persists is to determine the effect of different types of solids on *β*_m_ as well as its theoretical prediction without the applicability limit.

In this work, we present a theoretical model derived using an energy balance approach to predict *β*_m_. Particularly, our model considers the adhesion energy at the contact line in the vertical direction in addition to the horizontal direction^29^. The derived equation can predict *β*_m_ in a wide range of We (or Re) numbers for Newtonian liquid droplets on solid surfaces quantitatively without the use of arbitrary fitting parameters. We validate our model by comparing it to existing experimental data that employ micro- to millimetre-sized droplets^8, 17, 22, 30, 31^. In addition to these results, the transition point from the capillary regime to the viscous regime is theoretically determined.

## Theory

The energy conservation approach^9, 10^ considers both kinetic and surface energies prior to droplet impingement as well as surface energy and viscous dissipation after impingement. We now proceed to derive the theoretical equation expressing *β*_m_ as a function of *θ*_d_, Re, and We. Although some empirical and semi-empirical models exist in the literature^17, 22, 23, 26^, those models lack a quantitative prediction of *β*_m_. Recently, the importance of the work done by the adhesion force at the contact line, not only in the horizontal direction, but also in the vertical direction^29^ is revealed.

From an energy conservation standpoint, the contribution of the adhesion force in the vertical component must also be considered. Let *E*_kine_, *E*_surf_, *E*_grav_, *E*_sprd_, *E*_vis_, and *E*_def_ be the kinetic energy, initial surface energy, gravitational potential of the droplet, adhesion energy, viscous dissipation, and deformation energy after the impingement, respectively. Then, the following energy conservation holds:

1 $${E}_{{\rm{kine}}}+{E}_{{\rm{grav}}}+{E}_{{\rm{surf}}}={E}_{{\rm{sprd}}}+{E}_{{\rm{vis}}}+{E}_{{\rm{def}}},$$

where each term is expressed as follows:

2 $${E}_{{\rm{k}}{\rm{i}}{\rm{n}}{\rm{e}}}=\frac{1}{2}{\rho }_{{\rm{l}}}{V}_{0}{u}^{2},$$

3 $${E}_{{\rm{g}}{\rm{r}}{\rm{a}}{\rm{v}}}=\frac{{\rho }_{{\rm{l}}}g{h}_{{\rm{m}}}{V}_{0}}{2},$$

4 $${E}_{{\rm{s}}{\rm{u}}{\rm{r}}{\rm{f}}}=\pi {d}_{0}^{2}{\sigma }_{{\rm{l}}{\rm{g}}},$$

and

5 $${E}_{{\rm{s}}{\rm{p}}{\rm{r}}{\rm{d}}}=\pi {r}_{{\rm{m}}}^{2}{\sigma }_{{\rm{l}}{\rm{g}}}(1-\cos \bar{\theta })-\pi {r}_{{\rm{m}}}{h}_{{\rm{m}}}{\sigma }_{{\rm{l}}{\rm{g}}}\sin \bar{\theta },$$

6 $${E}_{{\rm{v}}{\rm{i}}{\rm{s}}}={\mu }_{{\rm{l}}}{(\frac{{u}_{{\rm{R}}}}{{h}_{{\rm{e}}{\rm{f}}{\rm{f}}}})}^{2}{V}_{0}{t}_{{\rm{m}}}.$$

7 $${E}_{{\rm{d}}{\rm{e}}{\rm{f}}}={S}_{{\rm{d}}{\rm{e}}{\rm{f}}}{\sigma }_{{\rm{l}}{\rm{g}}}$$

In Eqs (2–7), *ρ*_l_, *μ*_l_, *σ*_lg_, *V*_0_, *d*_0_, and *u* are the density of the liquid [kg m^−3^], viscosity of the liquid [Pa s], surface energy density of liquid [J m^−2^], initial droplet volume [m^3^], initial droplet diameter [m], and impinging velocity [m s^−1^] in the vertical direction, respectively. *r*_m_ (*i*.*e*., *d*_max_/2) is the maximum spreading radius [m], while *h*_m_, and *t*_m_ refer to the droplet height [m], and time [s] at which *r*_m_ is reached, respectively. *u*_R_ and *h*_eff_ are the radial velocity [m s^−1^] of the liquid along the solid surface after droplet impingement and the effective height [m] in the liquid film that is a distance from the wall, respectively. In Eq. (5), the first and second terms correspond to the adhesion energy in the horizontal and vertical directions at the contact line, respectively, and $\bar{\theta }$ is the simple averaged contact angle [°] of the static and dynamic contact angles. Of course, there exist a range of differences in droplet shapes ranging from a spherical cap to a flattened sphere. A quite low We number implies a spherical cap shape after impingement (gently depositing on the solid surface), whereas a large We number implies a flattened shape. Although it is well-known that the dynamic contact angle depends on We and Re, an exact determination of the contact angle is very difficult because of the scale differences that exist such as micro- and macro-contact angles^32^. In the present study, we assume that the exact value of the contact angle in the spreading process exists in the range between the contact angle at a quite low We value (*θ*_lowWe_) and that at a large We value (*θ*_highWe_). Here, with respect to *θ*_lowWe_, the fluid motion is negligible small in the quite low We situation at constant temperature. In such the case, we assume that the value of *θ*_st_ can be used as *θ*_lowWe_. Here, *θ*_st_ is determined by measuring the static contact angle of the droplet. Then, we consider *θ*_highWe_ to be *θ*_d_ at the maximum spreading diameter. Finally, the simple averaged values of *θ*_st_ and *θ*_d_ are used to give $\bar{\theta }$ = (*θ*_st_ + *θ*_d_)/2. Moreover, in the deformation term of Eq. (7), the exact evaluation of the surface area is also very difficult. Therefore, the deformed surface (*S*_def_) is defined as the harmonic average of the droplet surface of the spherical cap (*S*_cap_) and of the disk (*S*_disc_), given as

8 $${S}_{{\rm{def}}}=\frac{2{S}_{{\rm{cap}}}{S}_{{\rm{disc}}}}{{S}_{{\rm{cap}}}+{S}_{{\rm{disc}}}},$$

where

9 $${S}_{{\rm{cap}}}=\pi ({r}_{{\rm{m}}}^{2}+{h}_{{\rm{m}}}^{2}),$$

10 $${S}_{{\rm{disc}}}=\pi {r}_{{\rm{m}}}^{2}+2\pi {r}_{{\rm{m}}}{h}_{{\rm{m}}}.$$

In most energy conservation approaches^9, 10^, the initial impinging velocity (*u*) is used to evaluate the viscous dissipation. However, since shear stress occurs in the liquid film that spreads along the solid surface, the radial liquid velocity along the solid surface (*u*_R_) to evaluate the viscous dissipation term is more appropriate. When the liquid velocity reaches zero (*i*.*e*., kinetic energy is zero), the droplet diameter realizes its maximum spreading diameter. The treatment of the dissipation term is very difficult in this kind of analytical approach because the exact velocity distribution or profile is not known during the spreading process. However, it may be important to postulate a velocity profile for the evaluation of the viscous dissipation term. When the droplet impinges on the solid surface, the droplet shape initially becomes a bell shape owing to a recoil force from the solid surface, and then reaches the maximum spreading diameter caused by the surface tension^33^. This surface tension acts on the top of the bell-shaped droplet and pushes the liquid toward the solid surface, which then generates the radial liquid flow. This situation is like a wall jet along a solid surface^34–36^. Based on this assumption, the dissipation term can be evaluated using the velocity profile of wall jet.

The wall jet type velocity profile is non-linear and the peak of the velocity (*i*.*e*., maximum velocity) is located near the wall. From an experimental study of the wall jet^35^, it was found that the velocity peak is located at around one quarter of the effective height of the wall jet flow. However, in the case of the droplet impingement, the height is restricted by the droplet volume. If the liquid flow is confined by the wall and the liquid film surface (*i*.*e*., between parallel plates), however, the velocity profile becomes a parabolic shape where the peak of the velocity is located at half of the height. Thus, we postulate that the peak of the velocity profile in the case of the droplet impingement would be somewhere between these two situations of the wall jet flow and the parallel plate flow. Consequently, the effective height in Eq. (6) can be obtained by taking the harmonic average as follows:

11 $${h}_{{\rm{eff}}}\approx 2\frac{\frac{{h}_{{\rm{m}}}}{2}\frac{{h}_{{\rm{m}}}}{4}}{\frac{{h}_{{\rm{m}}}}{2}+\frac{{h}_{{\rm{m}}}}{4}}=\frac{{h}_{{\rm{m}}}}{3}.$$

**Figure 1:**
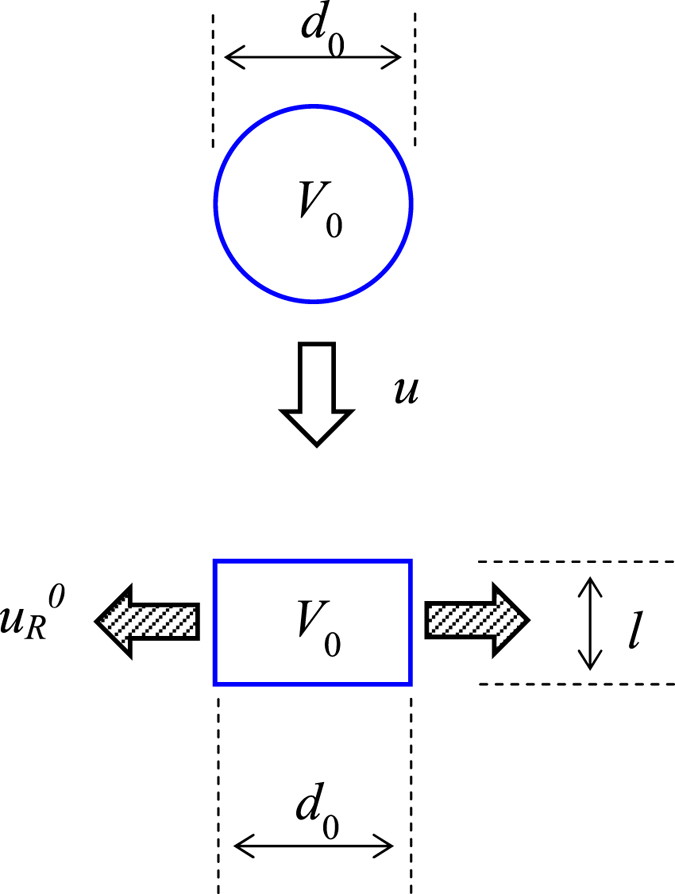
Simplified model to evaluate the radial velocity *u*_R_ in the viscous dissipation term. In the schematic, *u*, *V*_0_, *d*_0_, ${u}_{{\rm{R}}}^{0}$ and *l* represent the initial impinging velocity, droplet volume, droplet diameter, initial radial velocity and initial radial height.

Therefore, in the present model, the maximum velocity is characterized at the effective height of Eq. (11). As a next step, to evaluate *u*_R_ in Eq. (6) we need to calculate the initial radial velocity. However, the exact calculation is very difficult because the droplet shape is very complicated, as mentioned above. Thus, we estimate the initial radial-mean velocity ${u}_{{\rm{R}}}^{0}$, which is defined by assuming a cylindrically-shaped droplet of diameter *d*_0_ on the solid surface before spreading, as shown in Fig. 1. This assumption is used only for the analytical evaluation of the initial radial-mean velocity of the liquid. The droplet volume and velocity before impingement are denoted as *V*_0_ and *u*, respectively. At the moment of impingement on the solid surface, the liquid flows out from the cylindrical surface with an initial velocity of ${u}_{{\rm{R}}}^{0}$. Here, an equivalent height *l* of the cylindrical droplet is calculated as

12 $$l=\frac{\frac{\pi }{6}{d}_{0}^{3}}{\frac{\pi }{4}{d}_{0}^{2}}=\frac{2}{3}{d}_{0}.$$

Because of mass conservation,

13 $${\rho }_{{\rm{l}}}u\times (\frac{\pi }{4}{d}_{0}^{2})={\rho }_{{\rm{l}}}{u}_{R}^{0}\times \pi {d}_{0}l.$$

Combining Eqs (12) and (13) yields the following relation:

14 $${u}_{{\rm{R}}}^{0}=\frac{3}{8}u.$$

Because it is difficult to obtain a detailed velocity profile in the liquid, if the relationship between the maximum velocity *u*_max_ and the mean velocity *u*_mean_ satisfies *u*_max_ ≈ $2{u}_{{\rm{R}}}^{{\rm{mean}}}$, then ${u}_{{\rm{R}}}^{{\rm{\max }}\,,\,0}$ as characterised by the effective height of Eq. (11) becomes

15 $${u}_{{\rm{R}}}^{{\rm{\max }}\,,0}\approx 2{u}_{R}^{0}=\frac{3}{4}u.$$

As mentioned before, because the liquid velocity is zero when the droplet diameter reaches the maximum spreading diameter, the liquid velocity decreases from ${u}_{{\rm{R}}}^{{\rm{\max }}\,,0}$ to zero. For the sake of simplicity, the motion of the liquid film can also be characterised by a velocity that changes from ${u}_{{\rm{R}}}^{{\rm{\max }}\,,0}$ to zero. If the radial velocity in the radial direction of the spreading liquid film is known, the radial-mean velocity can be evaluated by

16 $${u}_{{\rm{R}}}=\frac{1}{{r}_{{\rm{m}}}-{r}_{0}}{\int }_{{r}_{0}}^{{r}_{m}}{u}_{{\rm{R}}}^{{\rm{\max }}}(r){\rm{d}}r,$$

where, *r* represents the spreading radius of liquid film, and ${u}_{{\rm{R}}}^{{\rm{\max }}}(r)$ represents the maximum velocity that changes from ${u}_{{\rm{R}}}^{{\rm{\max }}\,,0}$ at *r* = *r*_0_ to zero at *r* = *r*_max_. Again, because it is difficult to determine the exact function of ${u}_{{\rm{R}}}^{{\rm{\max }}}(r)$ in the case of droplet impingement, we assume that ${u}_{{\rm{R}}}^{{\rm{\max }}}(r)$ linearly decays with respect to the spreading radius as (*r*_m_ − *r*)${u}_{{\rm{R}}}^{{\rm{\max }}\,,0}$/(*r*_m_ − *r*_0_). Eventually, *u*_R_ of Eq. (16) can be calculated as 3 *u*/8 in the present study. Then, in the viscous dissipation term (Eq. (6)), *t*_m_ is the time when the kinetic energy *E*_kine_ is completely converted into the adhesion energy *E*_sprd_, the viscous dissipation *E*_vis_ and so on. Therefore, *t*_m_ is given by *r*_m_/*u*. The viscous dissipation term can be calculated by substituting *t*_m_ = *r*_m_/*u*, Eq. (11) and the relation of *u*_R_ = 3 *u*/8 into Eq. (6) as follows:

17 $$\begin{array}{ccc}{E}_{{\rm{v}}{\rm{i}}{\rm{s}}} & = & {\mu }_{{\rm{l}}}{(\frac{{u}_{{\rm{R}}}}{{h}_{{\rm{e}}{\rm{f}}{\rm{f}}}})}^{2}{V}_{0}{t}_{{\rm{m}}}\\  & = & {\mu }_{{\rm{l}}}\frac{81}{64}\frac{u{r}_{{\rm{m}}}}{{h}_{{\rm{m}}}^{2}}{V}_{0}\end{array}.$$

Finally, by substituting Eqs (2–5), (7) and (17) into Eq. (1), we arrive at the following equation:

18 $$\frac{{\rm{W}}{\rm{e}}}{3}-\frac{27}{16}\frac{{r}_{0}^{2}}{{h}_{{\rm{m}}}^{2}}{\beta }_{{\rm{m}}}\frac{{\rm{W}}{\rm{e}}}{{\rm{R}}{\rm{e}}}-(1-\cos \bar{\theta }){\beta }_{{\rm{m}}}^{2}+\frac{{h}_{{\rm{m}}}}{{r}_{0}}{\beta }_{{\rm{m}}}\sin \bar{\theta }+\frac{2}{3}\frac{{\rho }_{{\rm{l}}}\,g{r}_{0}{h}_{{\rm{m}}}}{{\sigma }_{{\rm{l}}{\rm{g}}}}+4-\frac{{S}_{{\rm{d}}{\rm{e}}{\rm{f}}}}{\pi {r}_{0}^{2}}=0.$$

In this equation, *h*_m_ is calculated as (see Supplementary Information)

19 $${h}_{{\rm{m}}}=\frac{2Af}{1+\frac{\pi }{{A}^{2}}f{\beta }_{{\rm{m}}}^{2}}{r}_{0}$$

20 $$f=\sqrt[3]{\frac{3}{\pi }+\sqrt{{(\frac{3}{\pi })}^{2}+\frac{{\beta }_{{\rm{m}}}^{6}}{{A}^{6}}}}+\sqrt[3]{\frac{3}{\pi }-\sqrt{{(\frac{3}{\pi })}^{2}+\frac{{\beta }_{{\rm{m}}}^{6}}{{A}^{6}}}},$$

21 $$A={(\frac{4\pi }{3})}^{1/3}.$$

In Eq. (18), the first, second, and fifth terms represent the non-dimensional kinetic energy $({E}_{{\rm{kine}}}^{\ast })$, the viscous dissipation $({E}_{{\rm{vis}}}^{\ast })$, and the gravitational potential $({E}_{{\rm{grav}}}^{\ast })$ respectively, while the third and fourth terms combined represent the adhesion energy $({E}_{{\rm{sprd}}}^{\ast })$. The sixth and seventh terms represent the initial surface energy $({E}_{{\rm{s}}{\rm{u}}{\rm{r}}{\rm{f}}}^{\ast })$ and the surface energy of the deformed surface $({E}_{{\rm{def}}}^{\ast })$, respectively. By using the definition of the Ohnesorge number (Oh = *μ*_l_/(*ρ*_l_ *d*_0_ *σ*_lg_)^1/2^ = We^1/2^Re^−1^), Eq. (18) can be solved for We^1/2^.

From Eq. (18), we can derive two limiting solutions for the capillary and viscous regions. In the capillary region, the viscous dissipation is negligible. Thus, we obtain the following relation:

22 $${\rm{W}}{\rm{e}}=3{\beta }_{{\rm{m}}}^{2}(1-\cos \bar{\theta })-\frac{3{h}_{{\rm{m}}}}{{r}_{0}}{\beta }_{{\rm{m}}}\sin \bar{\theta }-\frac{2{\rho }_{{\rm{l}}}g{r}_{0}{h}_{{\rm{m}}}}{{\sigma }_{{\rm{l}}{\rm{g}}}}-12+\frac{3{S}_{{\rm{d}}{\rm{e}}{\rm{f}}}}{\pi {r}_{0}^{2}}.$$

From this relation, we obtain a scaling law of *β*_m_ ∝ We^1/2^. In the viscous region, on the other hand, the kinetic energy and the viscous dissipation dominate, which leads to the following relation derived from Eq. (18):

23 $${\rm{Re}}\approx \frac{81}{16}{(\frac{1}{2Af}+\frac{\pi {\beta }_{{\rm{m}}}^{2}}{2{A}^{3}})}^{2}{\beta }_{{\rm{m}}}.$$

This relation gives rise to a scaling law of *β*_m_ ∝ Re^1/5^ ^6, 12^.

## Methods

In this work, we used two liquids – purified water (Wako Pure Chemical Industries, Ltd., Osaka, Japan) and pure ethanol (99.5% pure, Kenei Pharmaceutical Co. Ltd., Osaka, Japan) in order to understand droplet impingement for high- and low-surface energy liquids, whose densities (*ρ*), dynamic viscosities (*μ*), and surface tensions (*σ*_lg_) are given as follows: *ρ*_w_ = 998.2 kg m^−3^, *μ*_w_ = 10^−3^ Pa s, *σ*_lg,w_ = 72.8 × 10^−3^ N m^−1^, *ρ*_etha_ = 789.2 kg m^−3^, *μ*_etha_ = 1.2 × 10^−3^ Pa s, and *σ*_lg,etha _= 21.1 × 10^−3^ N m^−1^. The solids used were silicone rubber (SR) and polycarbonate (PC), which were 30 mm × 30 mm in size and 1 mm thick. Using an optical profilometer (NewView 5032, Zygo), we measured the mean values of the surface roughness (R_a_) to be 0.109 μm and 0.015 μm for SR and PC, respectively. We released 1.1-µL droplets using a microsyringe from ten different heights, *z* = 1.5 to 700 mm. The droplet can be regarded as a free-falling object in all experiments. A high-speed video camera (HX-5, NAC image technology, Ltd., Japan) with a microscope (Leica Microsystems, Wetzlar, Germany), captured images of the droplet behaviour after striking the solid surfaces; the frame rate is 20,000 fps. We measured the impinging velocity *u*, droplet diameter *d*_0_, and the maximum spreading diameter *d*_max_ based on the captured images. The values of We in our experiments ranged from 0.3 to 230. For high We number conditions, we used existing experimental data^8, 17, 22, 30, 31^ to verify the present model. All experiments were performed three times and conducted within temperature and humidity ranges of 21.0–23.0 °C and 51.0–55.0%, respectively.

## Results and Discussion

**Figure 2:**
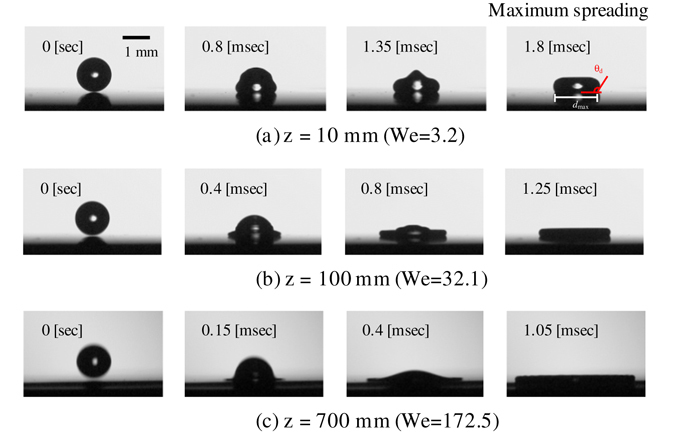
Images of a water droplet impinging on silicone rubber at varying initial drop heights. Images whose drops initially possess (**a**) We = 3.2 (*z* = 10 mm), (**b**) We = 32.1 (*z* = 100 mm), (**c**) We = 172.5 (*z* = 700 mm). Time progresses from the left image to the right image, where the far-right column of images represents drops exhibiting maximum spreading.

Figure 2 shows images of the water droplet impinging on the SR substrate from varying heights of *z* = 10, 100 and 700 mm, where the time advances from the left image to the right image. As the time advances, the droplet deforms and the droplet diameter reaches its maximum spreading state. From these images, it can be seen that the droplet shape initially becomes bell shaped and then reaches a disk shape at its maximum spreading diameter. The top surface of the bell-shaped droplet pushes the liquid down into the droplet.

**Figure 3:**
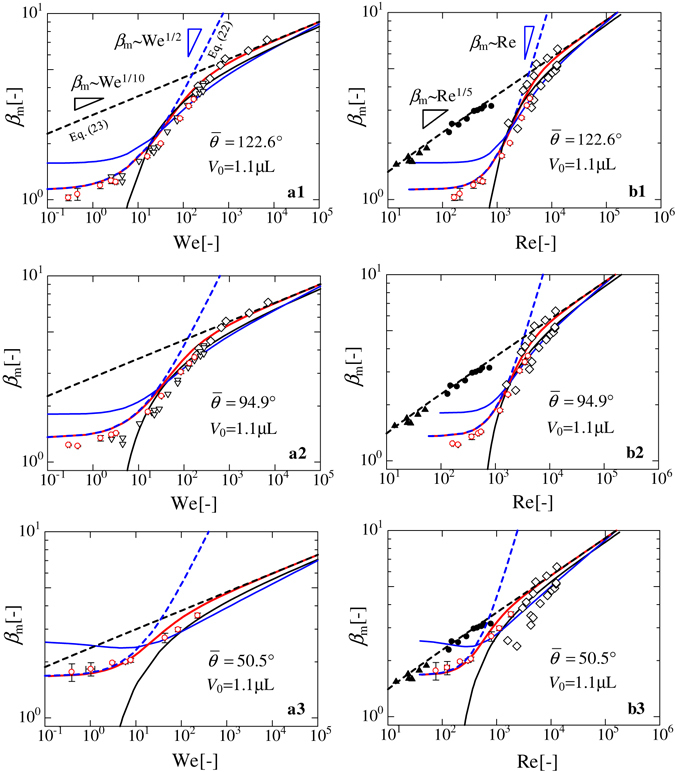
Relationships between *β*_m_ and non-dimensional numbers. (a1,a2 and a3) Plots of *β*_m_ *versus* We for purified water droplets on silicon rubber (SR) and on polycarbonate (PC), and ethanol on SR, respectively. (b1,b2 and b3) Plots of *β*_m_ *versus* Re for purified water droplets on SR and on PC, and ethanol on SR, respectively. The black and blue solid lines in each figure correspond to analytical results calculated by Roisman’s^27^ and Pasandideh-Fard *et al*.’s^10^ model, respectively. The blue and black dashed lines are the limiting solutions of the capillary (Eq. (22)) and viscous (Eq. (23)) regimes obtained by the present model. Here, *V*_0_ is the droplet volume and $\bar{\theta }$ is the averaged contact angle of *θ*_st_ and *θ*_d_. Red circles (⚬) represent the experimental data obtained in this study. White diamond (⬦) and inverted triangle (▿) markers represent water on an Al substrate and a superhydrophobic surface, respectively^17^. Black circle (⦁) and triangle (▴) markers represent existing experimental results for high-viscosity liquids (silicone oils) of *μ* = 20 mPa s and 300 mPa s, respectively^17^.

Figure 3-a1,a2 and a3 illustrate the relationships between *β*_m_ and We for purified water on SR and on PC, and pure ethanol on SR, respectively. The static contact angle and dynamic contact angle at the maximum spreading dimeter are *θ*_st_ = 116.6 [deg.] and *θ*_d_ = 128.2° for water on SR, *θ*_st_ = 87.0° and *θ*_d_ = 102.8° for water on PC, and *θ*_st_ = 34.5° and *θ*_d_ = 66.4° for ethanol on SR, respectively. Figure 3-b1,b2 and b3 illustrate the relationships between *β*_m_ and Re for the same combinations of liquids and solids shown in Fig. 3-a1,a2 and a3. In each figure, the solid red lines represent our model given by Eq. (18), while the solid black and blue lines correspond to existing models developed by Rosiman^27^, *β*_m_ = 0.87Re^1/5^ − 0.4Re^2/5^We^−1/2^, and Pasandideh-Fard *et al*.^10^, *β*_m_ = (We + 12)^1/2^/(3(1 − cos*θ*_d_) + 4(WeRe^−1/2^))^1/2^, respectively. We used the value of *θ*_d_ measured in our study for the model given by Pasandideh-Fard *et al*.^10^. The red circles denote experimental data that we collected in this study, while the white inverted triangles and diamond markers signify existing experimental data^17, 22^. Our model provides a better fit for the experimental data in each figure as compared to the two existing models, especially at low We numbers, where the two existing models deviate significantly from the experimental data. This result indicates the importance of considering adhesion energy in the vertical direction, in addition to the horizontal, in the capillary region. In each figure, the dashed blue and black lines are the limiting solutions corresponding to the capillary and viscous regions, respectively. Existing experimental data in Fig. 3-b1,b2 and b3 for high viscous liquids (black circles and triangle markers)^17^ are in good agreement with the limiting solutions in the viscous regime (Eq. (23)).

**Figure 4:**
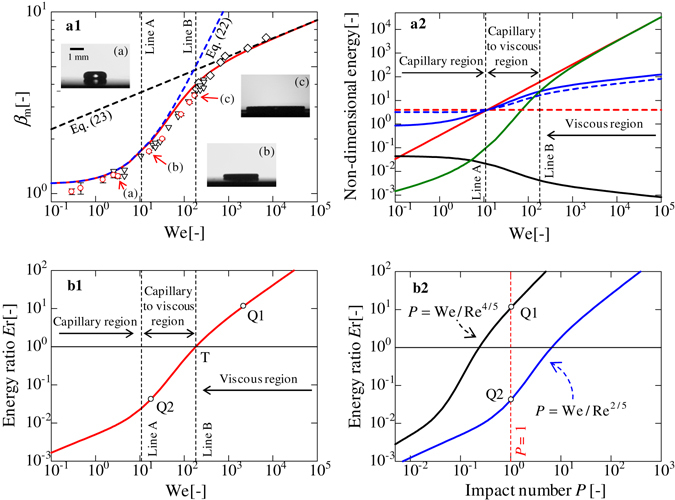
Energy transition from capillary to viscous regimes of a water droplet on SR. (a1) Plot of *β*_m_ *versus* We. Red circles (⚪) represent the experimental data obtained in this study. White diamonds (⬦) and inverted triangles (▿) represent water on an Al substrate and a superhydrophobic surface, respectively^17^. (a2) Plot of non-dimensional energy *versus* We. The solid red, black, blue, and green lines represent kinetic energy (${E}_{{\rm{kine}}}^{\ast }$ = We/3), gravitational potential (${E}_{{\rm{grav}}}^{\ast }$ = 2*ρ*_l_ *gr*_0_ *h*_m_/(3*σ*_lg_)), adhesion energy (${E}_{{\rm{sprd}}}^{\ast }$ = (1 − cos $\bar{\theta }$) ${\beta }_{{\rm{m}}}^{2}$ − (*β*_m_ *h*_m_/*r*_0_)sin $\bar{\theta }$), and viscous dissipation (${E}_{{\rm{vis}}}^{\ast }$ = 27${r}_{0}^{2}$ *β*_m_We/(16*h*_m_^2^Re)). The dashed red and blue lines represent initial surface energy (${E}_{{\rm{surf}}}^{\ast }$ = 4) and surface energy deformed surface (${E}_{{\rm{def}}}^{\ast }$ = *S*_def_/(π${r}_{0}^{2}$)). (b1) Plot of *E*_r_ *versus* We. (b2) Plots of *E*_r_ *versus* impact numbers *P* = We/Re^4/5^ and We/Re^2/5^.

Figure 4 depicts the transition between different droplet impingement conditions by taking a water droplet on SR as an example. Figure 4-a2 shows the distribution of kinetic energy ${E}_{{\rm{kine}}}^{\ast }$ (red line), adhesion energy ${E}_{{\rm{sprd}}}^{\ast }$ (blue line), viscous dissipation ${E}_{{\rm{vis}}}^{\ast }$ (green line), gravitational potential ${E}_{{\rm{grav}}}^{\ast }$ (black line), initial surface energy ${E}_{{\rm{surf}}}^{\ast }$ (red dashed line), and surface energy of deformed surface ${E}_{{\rm{def}}}^{\ast }$ (blue dashed line) with respect to the We number. In the region to the left of line A in Fig. 4-a2, which is the capillary region, ${E}_{{\rm{kine}}}^{\ast }$ is comparable to ${E}_{{\rm{sprd}}}^{\ast }$. Between lines A and B, which is the capillary-to-viscous region, the effect of viscous dissipation gradually appears until line B, at which point ${E}_{{\rm{vis}}}^{\ast }$ exceeds ${E}_{{\rm{sprd}}}^{\ast }$ in the viscous region. While the scaling law of *β*_m_ ∝ We^1/4^ ^17^ may potentially correspond to the intermediate region between lines A and B, we did not observe a scaling law of *β*_m_ ∝ We^1/4^ in our theoretical model. From Fig. 4-a2, the droplet condition of ${E}_{{\rm{vis}}}^{\ast }$ > ${E}_{{\rm{sprd}}}^{\ast }$ signals the onset of the viscous region. Thus, we can define the ratio of *E*_r_ = ${E}_{{\rm{vis}}}^{\ast }/{E}_{{\rm{sprd}}}^{\ast }$ (Eq. (A8) in Supplementary Information) in order to pinpoint the onset of the viscous region. The reason of the choice of ${E}_{{\rm{sprd}}}^{\ast }$ in *E*_r_ instead of ${E}_{{\rm{sprd}}}^{\ast }-{E}_{{\rm{surf}}}^{\ast }+{E}_{{\rm{def}}}^{\ast }$ is that the spreading process is directly affected by the wetting behaviour. When *E*_r_ > 1, the droplet falls under the viscous region. Figure 4-b1 presents the relationship between *E*_r_ and We. The intersection of *E*_r_ = 1 and line B represents the transition point *T*. Figure 4-b2 displays the relationships between *E*_r_ and impact numbers *P* = We/Re^4/5^ and *P* = We/Re^2/5^ ^7, 17, 37^. Two types of impact numbers are displayed in the figure. Although there is no consensus with respect to *P*, *P* = 1 is generally accepted as the boundary separating the capillary and viscous regions. However, our model indicates that both values of *P* cannot predict the transition point *T*. Strictly speaking, the point Q1, which is evaluated by *P* = We*/*Re^4/5^, is in the viscous region after the point *T*, whereas the point Q2, which is evaluated by *P* = We/Re^2/5^, is in the intermediate region between lines A and B. Here, the values of *P* = We*/*Re^4/5^ and We/Re^2/5^ are 0.2 and 6.5, respectively, when *E*_r_ = 1.

**Figure 5:**
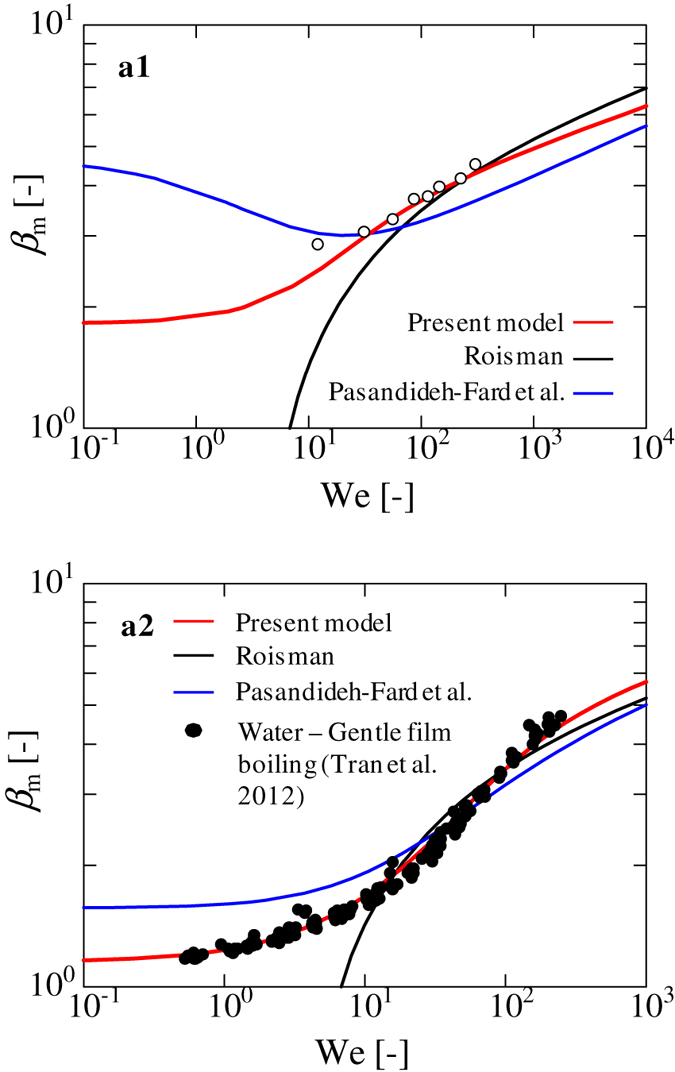
Comparison of our theory with existing experimental data. (a1) Plots of *β*_m_ *versus* We. The white circle represents ethanol on Al. The solid red, blue, and black lines represent analytical results from our theory, Pasandideh-Fard *et al*.^10^, and Roisman^27^, respectively. The droplet volume *V*_0_ is 8.2 μL. The values of $\bar{\theta }$ for ethanol used is 26.8° for our model. *θ*_d_ for Pasandideh-Fard *et al*. is 35.2°. (a2) Plots of *β*_m_ *versus* We for a water droplet on a heated substrate. The black circles represent the existing experimental data for water undergoing gentle film boiling on a heated silicon wafer^19^. The solid red, blue, and black lines represent the results of our model, and those of Pasandideh-Fard *et al*.^10^ and Roisman^27^, respectively. In our model, droplet volume is set to 6 μL. The same value of $\bar{\theta }$ as the water on SR case ($\bar{\theta }$ = 122.6°) is used in our experiment for each analytical result since the value of the dynamic contact angle was not reported. *θ*_d_ for Pasandideh-Fard *et al*. is also the same value of *θ*_d_ as the water on SR case (*θ*_d_ = 128.2°).

Figure 5-a1 and a2 compare our model with existing experimental data^19, 22^. Figure 5-a1 displays the results for ethanol on Al substrate. In this case, we used a droplet volume of 8.2 μL and reported value of *θ*_d_ = 35.2°. The static contact angle is not reported in the literature. Therefore, we estimate the static contact angle as *θ*_st_ = 18.3° where the ratio of *θ*_st_/*θ*_d_ = 0.52 evaluated by our experimental results in the case of ethanol on SR is used. Thus, the value of $\bar{\theta }$ is 26.8°. Our model provides a better agreement with the experimental data than the other two existing models. Figure 5-a2 offers additional results that illustrate the relationship between *β*_m_ and We for a water droplet undergoing gentle film boiling on a heated silicon wafer. “Gentle film boiling” describes a situation where the vapour layer is thick and prevents the liquid from touching the surface. However, this vapour layer has a non-uniform thickness, being thicker at the centre and thinner at the perimeter of the contact area^19, 38^. Thus, the liquid may come in contact with the surface of the solid at this perimeter, and the interaction between the solid and liquid at this perimeter would affect the spreading behaviour of the droplet. Our model captures the trend set by the experimental data, although, strictly speaking, we should also account for the temperature dependency of the physical properties. This result indicates that our model has the potential to predict and understand droplet impingement behaviour, including thermal effects. In this case, we used the same value of $\bar{\theta }$ used for the case of a water droplet on SR, owing to the lack of available contact angle data in literature^19^. In the case for the model given by Pasandideh-Fard *et al*.^9^, the value of *θ*_d_ = 128.2° is used.

**Figure 6:**
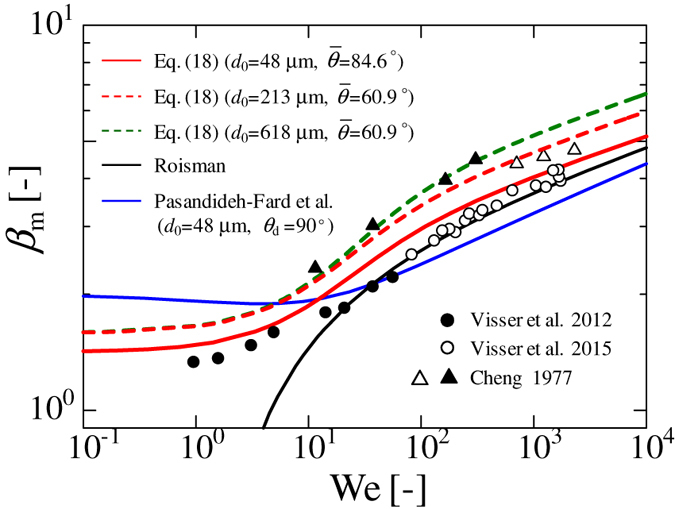
Comparison of our theory with existing experimental data for micro-sized water droplets at very high velocity. The black and white circles represent the existing experimental data for micro-sized water droplet impingement on a glass plate at very high velocity^8, 30^. The solid red line represents the theoretical results of Eq. (18) for *d*_0_ = 48 μm and $\bar{\theta }$ = 84.6°. The dashed red and green lines represent the analytical results of Eq. (18) for *d*_0_ = 213 μm and $\bar{\theta }$ = 60.9°, and for *d*_0_ = 618 μm and $\bar{\theta }$ = 60.9°, respectively. The solid blue and black lines represent the analytical results given by Pasandideh-Fard *et al*.^10^ and by Roisman^27^, respectively, for *d*_0_ = 48 μm and *θ*_d_ = 90°. The white (▵) and black (▴) triangles represent the existing experimental data for water droplets, *d*_0_ = 213 μm and 618 μm, respectively, on a coal surface. The reported static contact angle is 57°^31^. The white (⚬) and black (⦁) circles represent the existing experimental data for water droplets of *d*_0_ = 48 μm and 50 μm, respectively, on a glass plate. The contact angle was not reported.

We validated our model against existing experimental data for micro-sized droplet impingement on solid surfaces, as shown in Fig. 6. In this figure, both the white and black circles represent the existing experimental data from Visser *et al*. for micro-sized water droplets, *d*_0_ = 48 μm and 50 μm, respectively, on a glass plate^8, 30^. In the We <100 region, we used data reported in ref. 30, and in the We >100 region, we used data reported in ref. 8 because the accuracy of the data in the We >100 region in ref. 30 is reportedly poor. The solid red, blue, and black lines represent the theoretical results evaluated by our model, by Pasandideh-Fard *et al*.^10^, and by Rosiman^27^, respectively. From the image presented in ref. 30, we estimated the value of *θ*_d_ to be approximately 90°. The static contact angle *θ*_st_ is estimated as 79.2° using the ratio of *θ*_st_/*θ*_d_ = 0.88 which is averaged value of the water droplets on SR and PC in our experiment. Thus, the value of $\bar{\theta }$ is estimated as 84.6°. Although the result by Roisman shows good agreement with the experimental data in the high We number region, the results demonstrate that our model shows fairly good agreement with the experimental data from the low We number to the high We number region.

The white and black triangles represent the existing experimental data^31^ for water droplets, *d*_0_ = 213 μm and 618 μm, respectively, on a coal surface. The corresponding static contact angle is reported to be 57°. The dynamic contact angle is estimated as 64.8° using the ratio of *θ*_st_/*θ*_d_ = 0.88. Thus, the value of $\bar{\theta }$ is estimated as 60.9°, which we have adopted in our model (red and green dashed line). Our model was also successful in predicting the experimental data for droplets impinging on a coal surface.

## Conclusion

In this article, we have presented experimental and theoretical considerations of droplet impingement on solid surfaces. Our model accurately predicts the impinging behaviour of several kinds of liquids on various solid surfaces. According to the equations that we derived based on our theoretical considerations, *β*_m_ observes a scaling law of *β*_m_ ∝ We^1/2^ (∝Re) in the capillary region and *β*_m_ ∝ We^1/10^ (∝Re^1/5^) in the viscous region. In addition, the contribution of each energy component to the variation of *β*_m_ indicates that impact numbers, such as *P* = We/Re^4/5^ and *P* = We/Re^2/5^, cannot predict the transition point between the capillary and viscous regimes. Instead, our theory proposed using *E*_r_ instead of *P* to identify the boundary between the capillary and viscous regions. The present work, however, mainly considers a large-density ratio situation where the effect of the surrounding gas is negligible. To confirm the validity of our model, the low-density ratio situation must be considered because some vortex motions of the surrounding fluid may affect the impinging behaviour. In addition, we did not address non-Newtonian fluids in this study. To do so, the viscous term would have to be reconsidered to reflect the different expressions for the shear stress as compared to that of a Newtonian fluid. Nevertheless, the theoretical model that we presented in this paper has the potential of becoming a powerful tool to analyse droplet impingement behaviour. In particular, for inkjet droplets in precision engineering applications, such as soldering of electronics and microarrays for semiconductor components, our model can guide the development and precise fabrication of nano- and microstructures used in high-performance systems and devices.

## Electronic supplementary material

Derivation of some equations
