# Supplementary material for: Analytical consideration of liquid droplet impingement on solid surfaces
Source: Sci Rep. 2017 May 24;7:2362. doi: 10.1038/s41598-017-02450-4 (PMC5443818; doi:10.1038/s41598-017-02450-4)
Supplement: Supplementary file 1 — Derivation of some equations [file 41598_2017_2450_MOESM1_ESM.doc]

Analytical consideration of liquid droplet impingement on solid surfaces

Yukihiro Yonemoto^1)*^, Tomoaki Kunugi^2)^

^1^Priority Organization for Innovation and Excellence, Kumamoto University, 2-39-1, Kurokami, Chuo-ku, Kumamoto-shi, Kumamoto, 860-8555, Japan

^2^Department of Nuclear Engineering, Kyoto University, C3-d2S06, Kyoto Daigaku-Katsura, Nishikyo-ku, Kyoto, 615-8540, Japan

^*^Corresponding author: yonemoto@mech.kumamoto-u.ac.jp

**Derivation of *h*_m_ (Equation (19))**

Figure A1 displays the relationship between the maximum spreading diameter *d*_m_ and the droplet height at *d*=*d*_m_ (*h*_m_) for various combinations of liquids and solids. The images labelled (a)–(d) are examples of water droplets impinging on an SR substrate from heights of *z* = 2, 10, 50, and 100 mm, respectively. The plot illustrates the results of modelling the droplet as a portion of a sphere (solid black line) and a disc shape (solid blue line); where these lines are respectively obtained by solving *V*_cap_ = π*h*(*h*^2^/6+*r*^2^/2) and *V*_disc_ = π*r*^2^*h*. From these results, it is found that most experimental data exist between these two theoretical lines.


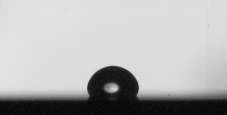

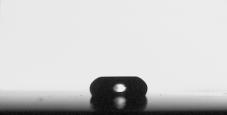

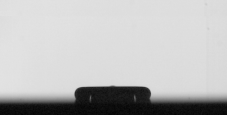

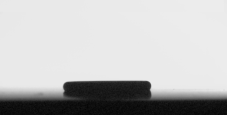


Droplet height *h*_m_ at *d*=*d*_m_ [m]

Water on SR

Water on PE

Water on PC

Ethanol on SR

*V*_0_ = 1.1 μL

1 mm

*z* = 2 mm

*z* = 10 mm

*z* = 50 mm

*z* = 100 mm

(a)

(b)

(c)

(d)

(a)

(b)

(c)

(d)

Maximum spreading diameter *d*_m_ [m]

**Fig. A1 | Relationship between maximum droplet diameter (*d*_m_) and droplet height (*h*_m_).**

The plot shows the experimental results for the droplet height at *d*=*d*_m_ (*h*_m_) as a function of the *d*_m_ of water droplets (volume *V*_0_ = 1.1 μL) impinging on silicone rubber (SR) (○), polyethylene (PE) (), and polycarbonate (PC) (), as well as that of ethanol droplets (*V*_0_ = 1.1 μL) on SR (). (**a**–**d**) Images of a water droplet at its maximum diameter on an SR substrate, released from various heights of (**a**) 2, (**b**) 10, (**c**) 50 and (**d**) 100 mm.

Here, the droplet shape varies from that of a spherical cap to a more flattened shape depending on the height from which the droplet is released. However, it is very difficult to obtain a universal analytical equation that can model the entire range of possible droplet shapes and to calculate the droplet volume even if the value of the contact angle can be predicted. Therefore, we assume that the diameter and the height of a droplet that varies from the spherical cap to the flattened shape can be evaluated by the following relations:

$V_{0}=\pi h_{cap}\left(\frac{h_{cap}^{2}}{6}+\frac{r_{m}^{2}}{2}\right)$, (A1)

$V_{0}=\pi r_{m}^{2}h_{disc}$, (A2)

and the height *h*_m_ is evaluated by the following harmonic average of *h*_cap_ and *h*_disc_:

$h_{m}=2\frac{h_{cap}h_{disc}}{h_{cap}+h_{disc}}$. (A3)

From equations (A2) and (A3), the following relation is obtained:

$h_{cap}=\frac{h_{m}V_{0}}{2V_{0}-\pi r_{m}^{2}h_{m}}$. (A4)

Next, the substitution of equation (A4) into equation (A1) yields

$X^{3}+3r_{m}^{2}X-\frac{6V_{0}}{\pi}=0$, (A5)

$X=\frac{h_{m}V_{0}}{2V_{0}-\pi r_{m}^{2}h_{m}}$. (A6)

Finally, equation (A5) is exactly solved from the solution of *x*^3^+*ax*+*b*=0 as

. (A7)

Therefore, equation (19) in the manuscript is ultimately derived by considering equations. (A5), (A6) and (A7). In Fig. A1, the solid red line is obtained by solving equation (19).

**Transition point from the capillary to viscous regime**

From the energy distribution shown in Figure 4-a2, the transition from the capillary regime to the viscous regime occurs when *E*_sprd_^*^ = *E*_vis_^*^. In other words, the transition point can be evaluated using the ratio of *E*_sprd_^*^ and *E*_vis_^*^:

$E_{r}=\frac{E_{vis}^{*}}{E_{sprd}^{*}}=\frac{27r_{0}^{2}}{16h_{m}^{2}\left[\beta_{m}(1-\cos\bar{\theta})-\frac{h{}_{m}}{r_{0}}\sin\bar{\theta}\right]}\frac{We}{Re}$. (A8)

If *E*_r_ is greater than unity, the droplet impingement is in the viscous regime. If *E*_r_ is less than unity, the droplet impingement is in the capillary regime. Weber number at the transition point can be obtained by considering the following relation that is derived using *E*_sprd_^*^ = *E*_vis_^*^:

$We=\left\{\frac{16h_{m}^{2}}{27r_{0}^{2}Oh}\left[\beta_{m}(1-\cos\bar{\theta})-\frac{h_{m}}{r_{0}}\sin\bar{\theta}\right]\right\}^{2}$. (A9)

We can compute the value of We and *β*_m_ using Eqs. (A9) and (18).
